# Pharmacological Preconditioning Using Diazoxide Regulates Store-Operated Ca^2 +^ Channels in Adult Rat Cardiomyocytes

**DOI:** 10.3389/fphys.2019.01589

**Published:** 2020-01-14

**Authors:** Raúl Sampieri, Eridani Fuentes, Elba D. Carrillo, Ascención Hernández, María C. García, Jorge A. Sánchez

**Affiliations:** Departamento de Farmacología, Centro de Investigación y de Estudios Avanzados del IPN, Mexico City, Mexico

**Keywords:** Orai channels, STIM, SOCs, cardiomyocyte, pharmacological preconditioning, diazoxide, ROS, ischemia–reperfusion

## Abstract

Voltage-dependent Ca^2+^ channels and store-operated Ca^2+^ channels (SOCs) are the major routes of Ca^2+^ entry into mammalian cells. Previously, we reported that pharmacological preconditioning (PPC) leads to a decrease in the amplitude of L-type calcium channel current in the heart. In this study, we examined PPC-associated changes in SOC function. We measured adult cardiomyocyte membrane currents using the whole-cell patch-clamp technique, and we evaluated reactive oxygen species (ROS) production and intracellular Ca^2+^ levels in cardiomyocytes using fluorescent probes. Diazoxide (Dzx) and thapsigargin (Tg) were used to induce PPC and to deplete internal stores of Ca^2+^, respectively. Ca^2+^ store depletion generated inward currents with strong rectification, which were suppressed by the SOC blocker GSK-7975-A. These currents were completely abolished by PPC, an effect that could be countered with 5-hydroxydecanoate (5-HD; a selective mitochondrial ATP-sensitive K^+^ channel blocker), an intracellular mitochondrial energizing solution, or Ni^2+^ [a blocker of sodium–calcium exchanger (NCX)]. Buffering of ROS and intracellular Ca^2+^ also prevented PPC effects on SOC currents. Refilling of intracellular stores was largely suppressed by PPC, as determined by measuring intracellular Ca^2+^ with a fluorescent Ca^2+^ indicator. These results indicate that influx of Ca^2+^ through SOCs is inhibited by their ROS and Ca^2+^-dependent inactivation during PPC and that NCX is a likely source of PPC-inactivating Ca^2+^. We further showed that NCX associates with Orai1. Down-regulation of SOCs by PPC may play a role in cardioprotection following ischemia–reperfusion.

## Introduction

Severe damage to heart muscle caused by ischemia and reperfusion (I/R) has been shown to involve Ca^2+^ overload during reperfusion (Murphy and Steenbergen, 2008). Mitochondria are key participants and regulators of myocardial injury during I/R. I-induced mitochondrial stress leads to the onset of mitochondrial permeability transition pore (MPTP) opening, release of cytochrome c through MPTPs, and, eventually, consequent cell death during reperfusion (Lesnefsky et al., 2017).

Ischemia and reperfusion injury can be mitigated by preconditioning with brief periods of ischemia (Murry et al., 1986) or by pharmacological preconditioning (PPC) with agents, such as diazoxide (Dzx), that open mitochondrial ATP-sensitive K^+^ channels (mitoKATPs) (Garlid et al., 1997; Pain et al., 2000). Blockade of mitoKATP channels prevents both ischemic preconditioning and PPC (Ardehali and O’Rourke, 2005; Halestrap et al., 2007). Pharmacological activation of mitoKATPs depends on reactive oxygen species (ROS) generated in mitochondria during preconditioning, which act to prevent mitochondrial Ca^2+^ overloading and MPTP opening (Lesnefsky et al., 2017). In addition to mitochondrial channels, L-type calcium channels play a role in PPC in adult rat cardiomyocytes (González et al., 2010). PPC reduces both Cav1.2 channel current amplitudes and action-potential produced surges in myoplasmic Ca^2+^ concentrations, which then attenuates I/R-induced damage (González et al., 2010). There has been little exploration of potential PPC-induced changes in the functions of other types of channels involved in Ca^2+^ homeostasis.

Ca^2+^ ions are critical mediators in cardiac excitation–contraction coupling, the process that enables the heart chambers to contract and relax. The development of contraction depends on a rise in myoplasmic Ca^2+^ concentration. During the cardiac action potential, the Ca^2+^ influx through depolarization-activated Cav1.2 channels in the cell’s plasma membrane triggers the release of Ca^2+^ from the sarcoplasmic reticulum (SR) (Bers, 2002). Subsequently, to enable relaxation, Ca^2+^ is removed from the cytosol via four transporters, namely the SR Ca^2+^ pump. the sarcolemmal Ca^2+^ ATPase, the sodium–calcium exchanger (NCX; Hinata et al., 2002), and the mitochondrial Ca^2+^ uniporter (Bers, 2002). Because NCX is electrogenic, it can transport Ca^2+^ out of or into (reverse mode) the myocyte, depending on the membrane potential (Bers, 2002). In the adult mammalian heart, the predominant two Ca^2+^ influx pathways are Cav1.2 channels and NCXs.

Sodium–calcium exchanger-mediated Ca^2+^ influx has a dual role in ischemic settings. That is, it has a deleterious role during I/R-induced damage as well as a beneficial role via triggering cardioprotective mechanisms during ischemic preconditioning (Castaldo et al., 2017). The mechanism by which NCX promotes ischemic tolerance is not yet known.

In non-excitable cells, Ca^2+^ influx is mediated primarily by a family of proteins known as store-operated Ca^2+^ channels (SOCs). As implied by their name, SOCs respond to intraluminal Ca^2+^ reductions in the endoplasmic reticulum. The stromal interaction molecule (STIM) and Orai proteins have been identified as Ca^2+^ sensors and store-operated channels, respectively. Their main role is to replenish internal Ca^2+^ stores (Prakriya and Lewis, 2015). In excitable cells, such as cardiomyocytes, store-operated Ca^2+^ entry (SOCE) has also been described (Hunton et al., 2004).

Although the role of SOCs in the adult heart is not well defined, SOC blockade has been shown to enhance functional recovery of heart muscle tissues following I/R (Liu et al., 2006; Collins et al., 2013), suggesting that SOCE may play a role in Ca^2+^ overload. In this study, we tested the hypotheses that (1) SOCE is downregulated during PPC, (2) downregulation of SOCE results from SOCs inactivation, and (3) that inactivation involves NCX and mitoKATP channels. Because PPC involves mitoKATP channel opening and increased ROS levels (González et al., 2010), and Orai1, but not Orai3, channels are inactivated by ROS (Bogeski et al., 2010), we examined how PPC cardiomyocyte physiology is affected by the ROS scavenger *N*-acetylcysteine (NAC). SOCs are inactivated by Ca^2+^ (Zweifach and Lewis, 1995). Thus, to test whether intracellular (cytosolic) Ca^2+^ concentration ([Ca^2+^]_i_) elevation contributes to SOC inactivation, in addition to ROS, we buffered myoplasmic Ca^2+^ with a Ca^2+^ chelator. SOC activation has been reported to require actively respiring mitochondria and mitochondrial Ca^2+^ uptake can be maintained when mitochondria are induced to remain in an energized state (Gilabert and Parekh, 2000; Glitsch et al., 2002). To test whether decreased SOC activation in PPC cardiomyocytes is related to de-energization of mitochondria, we examined whether the addition of a cocktail to the pipette solution that maintains mitochondria in an energized state restores SOC currents in PPC cardiomyocytes.

## Materials and Methods

### Subjects and Ethics

Experiments were conducted with male Wistar rats (300–350 g) employing protocols approved by the Division of Laboratory Animal Units and CINVESTAV-IPN, in compliance with federal law, federal statute, and *Consejo Nacional de Ciencia y Tecnología* regulations in Mexico. Rats were anesthetized with sodium pentobarbital (50 mg/kg, intraperitoneally), which was injected with sodium heparin (500 U/kg, intraperitoneally).

### Isolation of Ventricular Myocytes

Ventricular myocytes were isolated as described previously (Narasimhan et al., 2018), with slight modifications. In brief, adult rat hearts were mounted in a Langendorff apparatus and perfused for 5 min at 37°C with Ca^2+^-free Tyrode’s solution containing 136 mM NaCl, 5.4 mM KCl, 1 mM MgCl_2_, 10 mM HEPES, and 10 mM glucose. Unless otherwise stated, all chemicals were purchased from Sigma–Aldrich (St. Louis, MO, United States). Hearts were recirculated for ∼60 min using Tyrode’s solution supplemented with 70 U/mL type II collagenase (Worthington, Lakewood, NJ, United States) and 0.5 mg/100 mL type XIV protease. Ventricles were minced and shaken two to three times at 2 × *g* for 7 min in the same solution. Dislodged cells were filtered through a cell strainer (100 mm nylon BD Falcon, Fisher Scientific, Waltham, MA, United States) and centrifuged at 72 × *g* for 2 min. The pellet was re-suspended in Tyrode’s solution and the cardiomyocytes thus harvested were used immediately.

### Electrophysiology

We recorded membrane currents in dissociated adult rat ventricular myocytes using the whole-cell patch-clamp technique, as described previously (González et al., 2010). Currents were recorded using an Axopatch 200-A amplifier (Axon Instruments, Foster City, CA, United States). To measure membrane capacitance, 10 mV depolarizing pulses were applied. Current records lasting 100–300 s were digitized at a sampling interval of 120 ms via a Digidata interface (Axon Instruments, Foster City, CA, United States) at a 16-bit resolution. To measure the voltage dependence of membrane currents, ramps from +50 to −120 mv lasting 1 s were delivered every 10 s, and currents were sampled at 1-ms intervals. The holding potential (HP) was −80 mV. Data were analyzed using pCLAMP 8.0 (Axon Instruments, Foster City, CA, United States) and an in-house software. The standard pipette solution (pH 7.2) contained 137 mM cesium aspartate, 2 mM CsCl, 8 mM MgSO_4_, 1.8 mM MgCl_2_, 10 mM EGTA, and 15 mM HEPES. The bath solution (pH 7.4) contained 137 mM NaCl, 5.4 mM KCl, 1 mM MgCl_2_, 1.8 mM CaCl_2_, 10 mM glucose, 10 μM verapamil, 200 μM ouabain, and 10 mM HEPES. To deplete SR Ca^2+^ stores, we used the SR Ca^2+^-ATPase blocker thapsigargin (Tg) at a concentration of 2 μM from a 2-mM stock solution in dimethyl sulfoxide (DMSO). The ROS scavenger NAC was used at a concentration of 2 mM. NCX was blocked with 5 mM Ni^2+^ (Hinata et al., 2002). Orai1 channels were blocked with GSK-7975-A at a concentration of 10 μM that completely blocks Orai1/Orai3 channels (Derler et al., 2013).

Where indicated, the standard pipette solution was supplemented with a mitochondrial cocktail solution to maintain the mitochondria in an energized state (Gunter and Pfeiffer, 1990). This cocktail contained 2 mM pyruvic acid, 2 mM malic acid, 1 mM NaH_2_PO_4_, 0.5 mM cAMP, and 0.5 mM MgCl_2_. To observe the effect of intracellular Ca^2+^ on SOC inactivation, we added the cell-permeant Ca^2+^ chelator BAPTA-AM [1,2-Bis(2-aminophenoxy)ethane-*N*,*N*,*N*′,*N*′-tetraacetic acid tetrakis(acetoxymethyl ester) 30 μM, Molecular Probes/Thermo Fisher, Waltham, MA, United States, 30 min prior to electrophysiological recordings, as has been done previously (Yu et al., 2013).

Pharmacological preconditioning of cardiomyocytes was performed in Tyrode’s solution supplemented with 100 μM Dzx (Tocris Bioscience, Bristol, United Kingdom). Dzx was added from a 0.1-M stock solution in DMSO to achieve a final DMSO concentration <0.01%. At the concentration used in this study, Dzx is a selective opener of mitoKATP channels (Garlid et al., 1996). The selective mitoKATP channel inhibitor 5-hydroxydecanoate (5-HD; Hu et al., 1999) was used at a concentration of 100 μM. To characterize PPC effects on membrane currents, intact cardiomyocytes were incubated following the experimental protocols indicated in the figures and individual cardiomyocytes were patch-clamped to record whole-cell membrane currents. The experiments were performed at room temperature (23°C).

### Measurement of Cytosolic Ca^2+^

We measured [Ca^2+^]_i_ from single cardiomyocytes as described previously (González et al., 2010) with minor modifications. Briefly, cardiomyocytes were loaded with the cell-permeant fluorescent Ca^2+^ indicator Fura-2 acetoxymethyl ester (AM; Molecular Probes/Thermo Fisher, Waltham, MA, United States) for 45 min at room temperature. Fura-2 AM was diluted in Tyrode’s solution to a final concentration of ∼5 μM from a stock solution in DMSO containing 9 mM Fura-2 AM and 25% w/v Pluronic F127 (Molecular Probes/Thermo Fisher, Waltham, MA, United States). The cells were washed in Tyrode’s solution and attached to laminin-coated coverslips for 20–30 min at room temperature before performing [Ca^2+^]_i_ measurements. Ratiometric images of Fura-2 AM fluorescence were monitored with an Eclipse TE300 microscope (Nikon, Tokyo, Japan) equipped with a Polychrome V monochromator (TILL Photonics, Munich, Germany), allowing high speed changing between 340 and 380-nm excitation wavelengths. Fluorescence emissions were captured though a 510WB80 filter (Chroma Technology Corp., Bellows Falls, VT, United States) with an iXon EM + DU885 digital camera (Andor Technology, Belfast, United Kingdom). Image acquisition and ratio analysis were performed in Imaging Workbench 6.0 software (INDEC Biosystems, Los Altos, CA, United States). [Ca^2+^]_i_ was estimated as the Fura-2 AM fluorescence ratio (340/380). Ca^2+^ SR stores were depleted with caffeine (10 mM) and Tg (2 μM). *N*-Methyl-D-glucamine replaced extracellular Na^+^ equimolarly (Na^+^-free solution).

### Measurement of ROS Production

Reactive oxygen species levels were measured as described by Narasimhan et al. (2018) with the cell-permeant fluorescent probe 5-(and 6)-chloromethyl-20,70 dichlorodihydrofluorescein diacetate acetylester (CM-H2DCFDA; Molecular Probes/Thermo Fisher, Waltham, MA, United States). Fluorescence (505-nm excitation and 545-nm emission) was measured in arbitrary units (AU) for 30 ms in user-defined segments of cardiomyocytes within images acquired at 5-min intervals. Dzx-group cardiomyocytes were treated the same as those in the control group, except that they were pre-incubated with Dzx (100 μM) for 90 min. For each experimental condition, ROS measurements were normalized to initial fluorescence values and fitted to a straight line.

### PPC in Isolated Hearts

To assess protection against I/R, hearts previously perfused in Tyrode’s solution with or without Dzx (100 μM) for 90 min were subjected to global ischemia for 30 min, followed by 2 h of reperfusion with the control solution. Hearts were dyed, as described elsewhere (González et al., 2010), with 1% 2,3,5-triphenyltetrazolium chloride (TTC) for 10 min, fixed overnight with paraformaldehyde (4%), cut into 500-μm slices, and photographed with a digital camera attached to a microscope (Olympus, Center Valley, PA, United States). TTC produces colored precipitates in the presence of dehydrogenase activity, which is severely reduced or non-existent in necrotic cells.

### Co-immunoprecipitation of Orai1 and NCX

Membrane extracts from whole ventricles and ventricular cardiomyocytes were obtained. Isolated ventricular cardiomyocytes, preincubated in Tyrode or Dzx (PPC), were washed with ice cold PBS and sonicated (4 × 15 s) in buffer I containing (in mmol/L, pH = 7.5): 10 Tris–HCl, 1 Na^+^ vanadate, 1 phenylmethylsulfonyl fluoride (PMSF), 100 NaF, 1 EGTA, and a cocktail of protease inhibitors (1× ThermoFisher Scientific, Waltham, MA, United States). Buffer II containing (in mol/L, pH = 7.5): 10 Tris–HCl, 300 KCl, 1 Na^+^ vanadate, 1 PMSF, 100 NaF, 1 EGTA, 20% sucrose, and a cocktail of protease inhibitors (1× ThermoFisher Scientific, Waltham, MA, United States) was added and centrifugation at 10,000 × *g* for 10 min at 4°C followed. The supernatant was centrifuged at 100,000 × *g* for 1 h at 4°C. The pellet was washed and re-suspended in buffer III containing (in mmol/L, pH = 7.5): 50 Tris–HCl, 150 NaCl, 0.05% SDS, 1% TritonX-100, 0.5% deoxycholate with 0.5% CHAPS for IP:NCX1 and without CHAPS for IP:Orai1, and a cocktail of protease inhibitors (1× ThermoFisher Scientific, Waltham, MA, United States) and incubated for 30 min at 4°C. Whole ventricles were minced in liquid nitrogen, Buffers I and II were added (1v:1v) and the tissue was homogenized. Thereafter, an identical procedure used to obtain the membrane fraction from cardiomyocytes was followed. The membrane fraction from cardiomyocytes or ventricles was then used for immunoprecipitation experiments. Protein content was measured with Bradford assay. Solubilized proteins were pre-incubated with protein A/G-sepharose beads. 350–400 μg of precleared proteins were incubated with anti-NCX1 or anti-Orai1 antibodies overnight. Protein A/G-sepharose beads were added to the immuno-complex for 2 h. Control was performed by incubation with rabbit IgG. The beads were centrifuged, washed five times in PBS plus Tween 20 (0.15%), and warmed at 60°C for 10 min in Laemmli buffer to release the bound proteins. After SDS–PAGE and Western blotting, nitrocellulose membranes were blocked and treated with the first antibody against NCX1 (1:1000) or against Orai1 (1:500). Secondary antibodies were horseradish peroxidase conjugated anti-rabbit antibody (1:200,000; ThermoFisher Scientific, Waltham, MA, United States) or anti-mouse (1:400,000) horseradish peroxidase conjugated secondary antibody (ThermoFisher Scientific, Waltham, MA, United States) for experiments involving NCX1 and Orai1 primary antibodies, respectively. The blots were developed using a chemiluminescence detection system and were scanned at 600 dpi. The source of antibodies was: polyclonal anti-NCX1 antibody (Proteintech Group, Inc., Rosemont, IL, United States), and polyclonal anti-Orai1 (ThermoFisher Scientific, Waltham, MA, United States).

### Data Analysis

The data, which are expressed as means ± standard errors of the mean (SEMs), were tested for normal distribution, were analyzed with independent *t*-tests when two groups were compared or analyses of variance (ANOVAs), followed by multiple comparison Dunnett’s test, used to compare each of a number of treatments with a control. A significance criterion of *p* < 0.05 was used.

## Results

### Role of ROS and Ca^2+^-Dependent Inactivation in PPC Inhibition of SOCs

**FIGURE 1:**
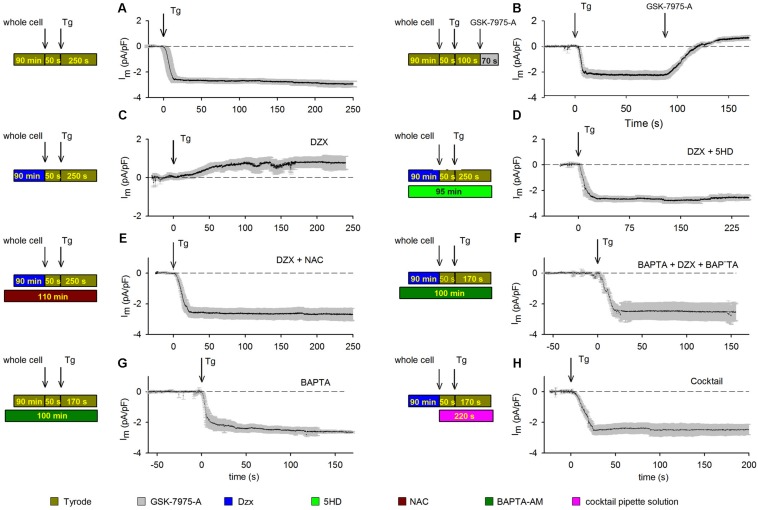
PPC inhibition of SOCs involves ROS and Ca^2+^-dependent inactivation. Visual summaries of experimental protocols are shown to the left of associated data graphs. Mean currents (±SEMs) are shown as a function of time and the HP was –80 mV in panels. **(A)** Membrane currents (*n* = 10) recorded from control cardiomyocytes treated with the SR Ca^2+^-ATPase blocker Tg (arrow). **(B)** The SOC inhibitor GSK-7975-A (arrow) blocked the inward current generated by Tg (*n* = 5); experiment otherwise as in panel **(A)**. **(C)** Membrane currents recorded in Dzx-induced PPC in cardiomyocytes (*n* = 13). **(D)** Blockade of mitoKATP channels with pre/concurrent 5-HD treatment reinstated the inward-current response to Tg in PPC cardiomyocytes (*n* = 7). **(E,F)** Pre/concurrent treatment of PPC cardiomyocytes with the ROS scavenger NAC (*n* = 5) **(E)** or with BAPTA-AM (*n* = 5) **(F)** prevented the effect of Dzx on Tg-induced inward currents. **(G)** Pre/concurrent BAPTA-AM treatment of control cardiomyocytes (no PPC) did not alter currents alone (*n* = 6). **(H)** Supplementing the pipette solution with a cocktail that keeps mitochondria energized in cardiomyocytes pretreated with Dzx also prevented the effect of Dzx on Tg-induced inward currents (±SEM, *n* = 6).

Depletion of cardiomyocyte intracellular Ca^2+^ stores with Tg led to the development of an inward current, which stabilized rapidly and was maintained throughout the recording period. The results from several experimental replicates are reported in Figure 1A. Membrane current values were normalized to unit capacitance to allow for comparisons among experiments. Mean membrane currents (±SEMs; *n* = 10) generated over time following addition of Tg are shown. To test whether SOCs may mediate the aforementioned currents, we blocked SOCs with 10 μM GSK-7975-A (Figure 1B). Depletion of intracellular Ca^2+^ stores with Tg produced inward currents (Figure 1A) that were completely inhibited by GSK-7975-A, followed by the development of a small outward current (Figure 1B).

We verified the ability of Dzx perfusion to produce PPC by exposing control and Dzx-pretreated hearts to severe ischemia (Supplementary Figure S1). Note that extensive areas of infarction are evident (light-colored areas) in an infarcted heart perfused with control solution before ischemia (representative cross-section in Supplementary Figure S1A). When an isolated heart was perfused with Dzx prior to ischemia, infarcted areas were greatly reduced (Supplementary Figure S1B). Similar results were obtained across experiments performed in triplicate.

Having confirmed our PPC model, we tested the effects of PPC on membrane currents. Tg-induced currents in cardiomyocytes were reversed with SOC blockade (Figures 1A,B). Dzx-induced PPC blocked Tg-generated currents; instead, we observed the development of a small outward current that did not inactivate (Figure 1C). In the presence of the mitoKATP channel inhibitor 5-HD, the PPC effect of Dzx was blocked and Tg-induced currents were re-enabled (Figure 1D), consistent with the hypothesis that mitoKATP channels are involved in the Tg response in PPC cardiomyocytes.

We tested the hypothesis that inhibition of SOC currents in PPC cardiomyocytes is due, at least in large part, to Orai1 inactivation by ROS. First, we verified that ROS production was indeed increased by Dzx treatment in cardiomyocytes under our experimental conditions (Supplementary Figure S2). Next, we found that in the presence of the ROS scavenger NAC, cardiomyocytes exhibit currents in response to Tg (Figure 1E) that were similar to that seen in control cardiomyocytes (Figures 1A,B), consistent with ROS involvement in the inhibition of SOCs in PPC cardiomyocytes.

In experiments testing whether PPC-related elevation of [Ca^2+^]_i_ plays a role in SOC inactivation, we found that Tg-induced inward currents were not inhibited by PPC when myoplasmic Ca^2+^ was buffered with BAPTA-AM (Figure 1F). BAPTA-AM alone had negligible effects on currents generated in non-PPC cardiomyocytes (Figure 1G). When a cocktail that keeps mitochondria in an energized state was added to the pipette solution, SOC-mediated currents were not inhibited in PPC cardiomyocytes (Figure 1H).

Data obtained with treatments illustrated in Figures 1B–H were compared with results from control experiments (Figure 1A) and analyzed for significant differences at *t* = 150 s after the addition of Tg. Only the currents from PPC cardiomyocytes (Figure 1C) and those from cardiomyocytes treated with GSK-7975-A SOCs blocker (Figure 1B) had a statistically significant difference (*p* < 0.001).

### Inhibition of SOCs by PPC at Different Potentials

**FIGURE 2:**
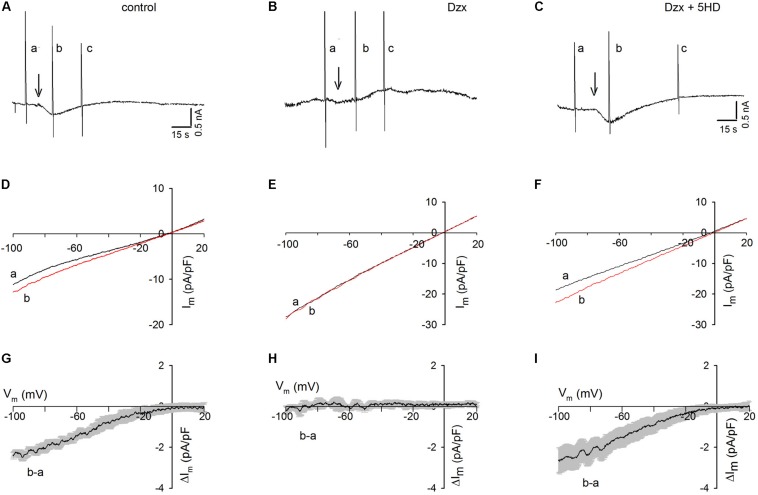
Dzx-induced PPC prevents SOC currents in cardiomyocytes upon application of voltage ramps. Black lines represent I–V curves during ramps before Tg; red lines represent I–V relationships during the first voltage ramp after Tg. **(A–C)** Continuous recordings of membrane currents under control **(A)**, Dzx pretreatment **(B)**, and Dzx + 5-HD pretreatment **(C)** conditions. Letters **a–c** indicate application of voltage ramps. Arrows indicate the addition of Tg. HP = –80 mV. **(D–F)** I–V curves correspond with the experiments illustrated above in panels **(A–C)**. Notice that the addition of Tg increased membrane currents **(D,F)**, as reflected by separation of the **a** and **b** curves, but had no effect when mitoKATP channels were opened by Dzx **(E)**. **(G–I)** Mean values (±SEM, *n* = 4–8) of Tg-generated currents **b–a** as a function of membrane potential from experiments performed exactly as those in panels **(D–F)**.

When three ramps (a, b, and c) were delivered to a control cardiomyocyte held at −80 mV (Figure 2A) and Tg was added after the first ramp, an inward current developed but, in contrast to that in cardiomyocytes held continuously at −80 mV (Figure 1A), the current was not maintained and decreased spontaneously. Consistent with the results seen in Tg-treated PPC cardiomyocytes in the previous experiment (Figure 1C), no inward currents were recorded in the PPC cardiomyocytes, and ramps had little effect (Figure 2B).

As shown in Figure 2C, in the presence of the mitoKATP channel inhibitor 5-HD, Tg administration resulted in the development of an inward current in PPC cardiomyocytes, which decreased spontaneously following the application of voltage ramps, as was seen in the non-PPC control cardiomyocytes (Figure 2A). The unsubtracted current–voltage (I–V) relationships during ramps of the experiments shown in Figures 2A–C are illustrated in Figures 2D–F. Estimates of the I–V relationships of Tg-activated SOC currents generated by subtracting currents during ramps before Tg from those recorded during ramps after Tg are summarized in Figures 2G–I.

In control experiments, Tg-induced SR Ca^2+^ depletion resulted in the activation of currents with strong inward rectification. In PPC cardiomyocytes, currents before and after Tg addition were almost identical, indicating complete inhibition of SOCs (Figure 2H), an effect that was blocked by 5-HD (Figure 2I).

### Ca^2+^-Dependent Inactivation of SOCs and Sodium–Calcium Exchanger

**FIGURE 3:**
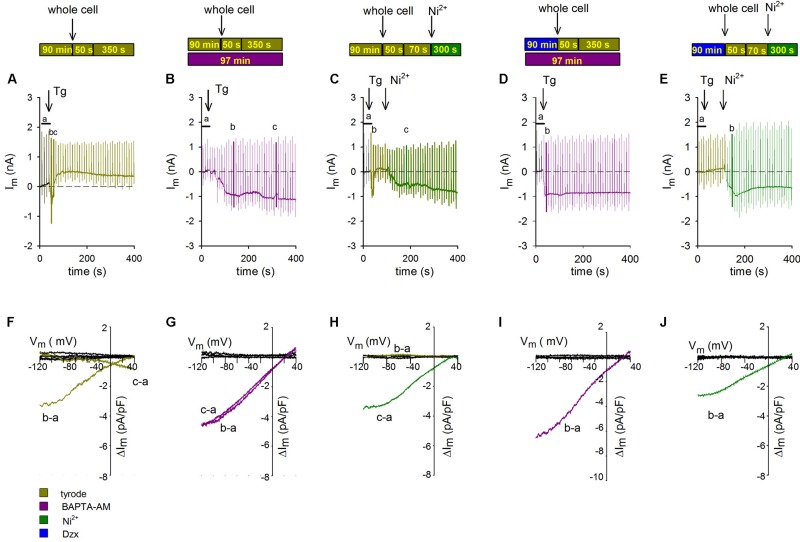
Inactivation of SOCs by voltage ramps is prevented by Ca^2+^ chelation with BAPTA-AM or NCX blockade with Ni^2+^. The experimental protocol employed for each experiment is summarized above the data associated with that experiment. Voltage ramps were delivered every 10 s with an HP of –80 mV. The additions of Tg and Ni^2+^ are indicated with arrows. **(A–C)** Representative continuous recordings of membrane currents in a control non-PPC cardiomyocyte **(A)**, a non-PPC cardiomyocyte preincubated with BAPTA-AM **(B)**, a non-PPC cardiomyocyte treated with Ni^2+^ **(C)**, a PPC cardiomyocyte preincubated with BAPTA-AM **(D)**, and a PPC cardiomyocyte treated with Tg and Ni^2+^ **(E)**. **(F–J)** I–V relationships of subtracted currents from the experiments illustrated in panels **(A–E)**. The average current upon control ramp application (indicated by the letter **a**) was subtracted from the currents upon the application of selected ramps, which are indicated by the letters **b** and **c** in panels **(A–E)**. Average control currents during ramps (black lines) were also subtracted from each individual control current.

To test whether changes in membrane potential during application of ramps would increase [Ca^2+^]_i_ in the vicinity of SOCs, and thereby promote SOC inactivation, voltage ramps were delivered every 10 s, first in Tyrode’s solution and then after addition of Tg (representative experiment shown in Figure 3A). Tg generated an inward current like those seen in non-PPC control cardiomyocytes (Figure 2A), but that current was not maintained and it turned outward in response to successive ramps. When the same ramp protocol was applied to cardiomyocytes previously incubated in the Ca^2+^ chelator BAPTA-AM, the inward current did not become inactivated, affirming the involvement of Ca^2+^ in the inactivation (Figure 3B).

To test whether the source of Ca^2+^ mediating SOC inactivation upon application of ramps was NCX, we used the NCX blocker Ni^2+^. In cardiomyocytes held at −80 mV and administered a protocol identical to that used in the experiments shown in Figures 3A,B, Tg-induced inward currents through SOCs that inactivated completely in response to successive ramps, after which a small outward current developed. The addition of Ni^2+^ restored SOC currents (representative experiment in Figure 3C). The I–V relationships of SOCs from the experiments shown in Figures 3A–C, determined by subtracting the average of currents exhibited upon ramp application before Tg addition from selected currents generated upon ramp application after Tg, are illustrated in Figures 3F–H. In non-treated, non-PPC cardiomyocytes, only the first ramp generated a clear inward-rectifying SOC current (Figure 3F; replication of the effect in Figure 2G). In the presence of BAPTA-AM, SOC currents were generated in non-PPC cardiomyocytes throughout the whole recording period (Figure 3G). Meanwhile, in non-PPC cardiomyocytes, the addition of Ni^2+^ reversed SOC current inactivation generated by successive ramps (Figure 3H). Values of subtracted control currents were confirmed to be close to zero at all voltages. Similar results were obtained in five additional experiments.

### Ca^2+^-Dependent Inactivation of SOCs in PPC Cardiomyocytes

Confirming the hypothesis that the Ca^2+^-dependent inactivation of SOCs is involved in the inhibition of currents upon ramp application in PPC cardiomyocytes, a ramp protocol applied in the presence of BAPTA-AM resulted in a non-inactivating inward currents (Figure 3D). In PPC cardiomyocytes, Tg-generated inward currents were absent (Figures 1C, 2B). SOC currents were restored in Tg-treated PPC cardiomyocytes following addition of Ni^2+^, suggesting that NCX may also participate in PPC-induced SOC inactivation (Figure 3E). The corresponding I–V relations of subtracted currents upon administration of ramps shown in Figures 3D,E are illustrated in Figures 3I,J, respectively. Similar results were obtained in five to eight additional experiments.

### I–V Relationships of SOC Currents in PPC Cardiomyocytes With Energized Mitochondria

**FIGURE 4:**
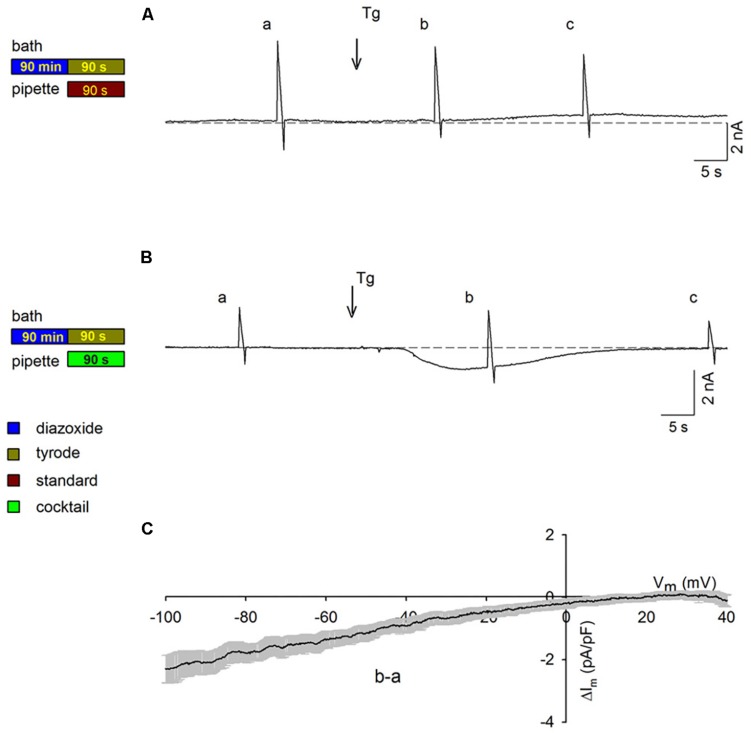
Activation of SOCs in PPC cardiomyocytes with energized mitochondria upon voltage ramp application. The experimental protocol employed for each experiment is summarized to the left of the data associated with that experiment. Ramps were applied at points **a**–**c** in the traces. Arrows show the addition of Tg; HP = –80 mV. **(A,B)** Representative continuous recordings of membrane currents in PPC cardiomyocytes (pretreated with Dzx) exposed to a standard pipette solution **(A)** or to a mitochondrion energizing cocktail pipette solution **(B)**. **(C)** Mean current values (±SEMs, *n* = 6) of SOC currents **b–a** as a function of membrane potential from experiments performed as in panel **(B)**.

Given our finding that PPC cardiomyocytes with energized mitochondria exhibited currents that were similar to those in control cardiomyocytes under quiescent conditions (Figures 1A,H), we assessed whether this finding would hold true when voltage ramps are applied. As shown in Figure 4A, during continuous recording of membrane currents in a PPC cardiomyocyte, performed using the standard pipette solution, no inward currents were observed after Tg administration upon voltage ramp application (consistent with the results in Figure 2B). Conversely, in a parallel experiment performed with a mitochondria energizing solution, in place of the standard pipette solution, inward SOC currents were observed after Tg administration upon voltage ramp application (representative experiment in Figure 4B). Depletion of intracellular Ca^2+^ stores generated an inward current with an amplitude and time course that were similar to those recorded in non-PPC cardiomyocytes (Figure 2A). Average subtracted currents (b, a) from several experimental replicates recorded using the energizing pipette solution are presented as a function of membrane potential upon the application of voltage ramps to PPC cardiomyocytes in Figure 4C. These currents exhibited inward rectification with an I–V relationship similar to that observed for non-PPC cardiomyocytes (Figure 2G).

### SOCs Are Not Active in PPC Cardiomyocytes, as Revealed by Fura-2 AM

**FIGURE 5:**
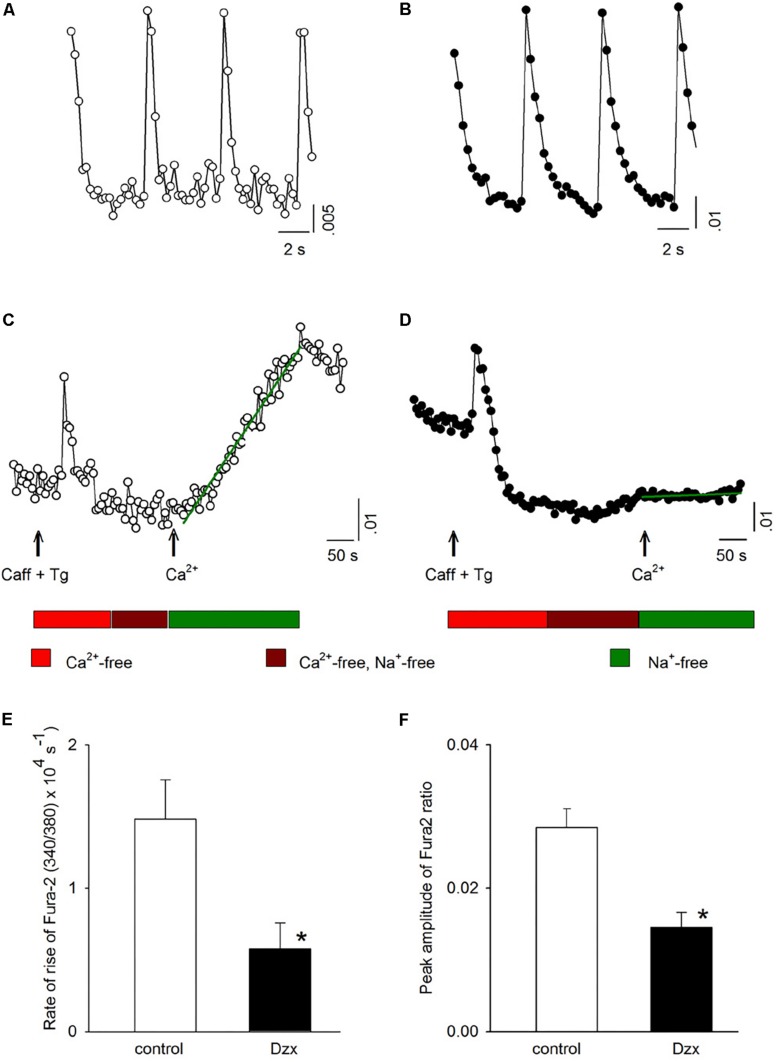
PPC with Dzx inhibits the influx of Ca^2+^ induced by SR depletion. **(A,B)** Ca^2+^ signals generated by action potentials in a control experiment **(A)** and in a PPC (Dzx-pretreated) cardiomyocyte **(B)**. **(C,D)** Intracellular Ca^2+^ measurements from the same respective cardiomyocytes whose data are shown in panels **(A)** and **(B)**, respectively, following the protocols illustrated below the associated data line graphs. The rate of the rise of intracellular Ca^2+^ associated with restoration of external Ca^2+^ was calculated on the basis of the slope of the straight lines superimposed over the data points following Ca^2+^ restoration (second arrows). **(E,F)** Mean values (±SEMs) of the rate of rise of intracellular Ca^2+^ **(E)** and corresponding intracellular Ca^2+^ level peaks **(F)** from replicates of the experiments shown in panels **(C)** and **(D)**; (*n* = 10–14), ^∗^*p* < 0.05.

To obtain further information on activation of SOCs in PPC cardiomyocytes, intracellular Ca^2+^ was measured. Figure 5A shows intracellular Ca^2+^ signals (visualized with Fura-2 AM and expressed as 340/380 ratios) associated with action potentials generated in a control cardiomyocyte. Figure 5C shows the change in intracellular Ca^2+^ for the same cardiomyocyte under conditions of emptied intracellular stores, as well as after Ca^2+^ was reintroduced into the external solution of the depleted cardiomyocyte, intracellular Ca^2+^ increased over time (Figure 5C; increase rate represented by the slope of the line fitted to the data points). When the same experiment was conducted in parallel in a PPC cardiomyocyte (Figures 5B,D), reintroduction of external Ca^2+^ produced only a subtle increase in intracellular Ca^2+^, indicating only a very low level of SOC activation (Figure 5D). Averaged results from several experiments like those shown in Figures 5C,D confirmed that the PPC-inducing Dzx treatment reduced the rate of intracellular Ca^2+^ rise (Figure 5E) and the peak amplitude of intracellular Ca^2+^ levels (Figure 5F) following reintroduction of external Ca^2+^, consistent with far fewer SOCs being open in PPC cardiomyocytes than in non-PPC control cardiomyocytes.

### Orai1 Co-immunoprecipitates With NCX1

**FIGURE 6:**
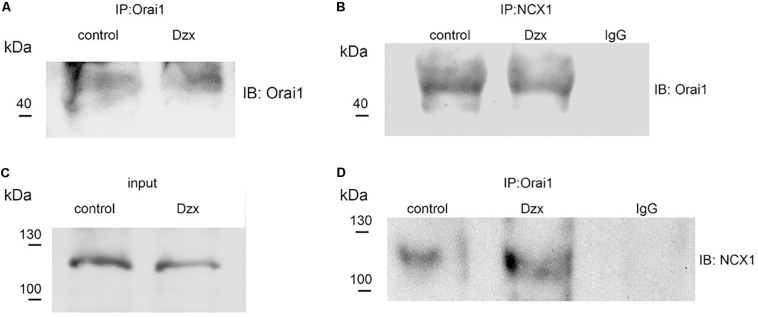
Orai1 associates with NCX1 in the adult rat heart. **(A,B)** Orai1 and NCX1 were immunoprecipitated from membrane fractions of control and PPC ventricles, respectively, and. immunoblotted with anti-Orai1 antibody. **(C)** NCX1 from membrane fractions of control and PPC ventricles was immunoblotted with anti-NCX1 antibody (input). **(D)** Orai1-immunoprecipitated proteins were immunoblotted with anti-NCX1 antibody.

We next examined whether Orai1 interacts with NCX1. NCX1 was immunoprecipitated from membrane fractions of control and PPC preparations with the NCX1 antibody, followed by immunoblotting for Orai1. Upon immunoprecipitating NCX1, a band with a molecular weight corresponding to Orai1 (∼50 kDa) was detected in the membrane fraction of control and PPC ventricles (Figure 6B). We verified with the specific Orai1 antibody that these fractions contained Orai1 (Figure 6A). Solubilized proteins from membrane fractions of control and PPC ventricles were immunoblotted to detect NCX1. On SDS-PAGE, the NCX1 protein in the adult heart appears as a predominant band of ∼120 kDa (Shigekawa and Iwamoto, 2001), as shown in Figure 6C. After immunoprecipitating with the Orai1 antibody and immunoblotting with the NCX1 antibody, NCX1 was detected in control and PPC ventricles, confirming Orai1–NCX1 interaction (Figure 6D). Non-specific binding was determined using anti-rabbit IgG which failed to pull down any Orai1/NCX1 immunoreactivity (Figures 6B,D). Similar results were obtained in three experimental replicates.

## Discussion

### PPC and SOCs in the Adult Heart

Myocardial cell death associated with I/R is related to major alterations in Ca^2+^ handling (Murphy and Steenbergen, 2008). Protection against injury can be achieved by PPC (Sato et al., 2000; Wang et al., 2001; Pasdois et al., 2008; González et al., 2010), which involves opening of mitoKATP channels through the use of drugs. We present here, the novel observation that SOCs in adult cardiomyocytes are largely inhibited by PPC based on SOC current recordings via the whole-cell patch-clamp technique and quantitation of the Ca^2+^ levels with the Fura 2 AM indicator.

Store-operated Ca^2+^ entry, a ubiquitous and functionally important Ca^2+^ homeostasis pathway first recognized in non-excitable cells, was found to be present and functional in excitable cells as well (Prakriya and Lewis, 2015). The SOC Ca^2+^ sensor STIM1 is expressed in skeletal muscle, where its absence leads to decreased force, accelerated fatigue, and eventual perinatal death in mice due to skeletal myopathy (Stiber et al., 2008). STIM1 is also expressed in heart muscle (Ohba et al., 2009; Domínguez-Rodríguez et al., 2015; Saliba et al., 2015; Zhao et al., 2015) and its cardiac-restricted ablation leads to ventricular dilatation (Collins et al., 2014), reduced left ventricular contractility, and decreased shortening of isolated cardiomyocytes (Parks et al., 2016). Orai1 channels are also expressed in the heart muscle as revealed by western blotting (Ohba et al., 2009; Horton et al., 2014; Domínguez-Rodríguez et al., 2015; Saliba et al., 2015). In zebra fish, Orai1 deficiency in cardiomyocytes results in severe heart failure, reduced ventricular systolic function, and bradycardia (Völkers et al., 2012). SOCs in the heart have also been associated with cardiac disease, playing a role in the development of hypertrophy in neonatal and adult cardiomyocytes (Hunton et al., 2002; Ohba et al., 2009; Luo et al., 2012; Ji et al., 2017). Both STIM1 and Orai1 have been implicated in hypertrophic cardiac growth regulation. In mice, STIM1 overexpression enhances Ca^2+^ influx following Ca^2+^ depletion and also leads to the induction of the fetal gene program and sudden cardiac death or hypertrophy (Correll et al., 2015). Meanwhile, Orai1 knockdown has a beneficial effect, preventing hypertrophy in neonatal cardiomyocytes (Voelkers et al., 2010).

In heart muscle, SOCE differs over the course of development. In rat neonatal cardiomyocytes, SOCE is prominent after SR depletion, as evidenced by SOC current recordings and myoplasmic Ca^2+^ measurements (Ohba et al., 2009; Ji et al., 2017). In adult cardiomyocytes, SOCE and STIM1 expression are less prominent (Luo et al., 2012), though SOC currents showing inward rectification and corresponding changes in intracellular Ca^2+^ have been observed under Tg-induced SR depletion conditions (Hunton et al., 2004; Saliba et al., 2015; but see Zhao et al., 2015). Our experiments showed the presence of inward rectifying SOC currents as a result of SR Ca^2+^ depletion in adult cardiomyocytes but do not provide direct evidence regarding the identity of the channel responsible for SOCE.

Recently, canonical transient receptor potential channels (TRPC) have been implicated in SOCE in neonatal cardiomyocytes (He et al., 2017) and TRPC3/6 channels specifically have been associated with I/R tissue damage (He et al., 2017). Although a role of TRPC channels in our experiments cannot be ruled out entirely, several lines of evidence suggest that Orai1 channels play a leading role in PPC-associated SOC current inactivation. Firstly, we found that SOC currents were completely suppressed by GSK-7975-A, which, at the concentration used, produces a complete block of Orai1 and Orai3 channels (Derler et al., 2013; Molnár et al., 2016) while most TRP channels (IC_50_ > 10 μM) remain unblocked at this concentration (Derler et al., 2013). Secondly, the TRP blocker SKF-96365 has no effect on membrane currents in Tg-treated adult cardiomyocytes (Domínguez-Rodríguez et al., 2015). Thirdly, our experiments revealed that SOC currents were inactivated by an elevation in ROS levels, and Orai1 channels are inhibited by oxidation (Bogeski et al., 2010). Several members of the TRP channel family are activated by ROS, including TRPC3 and TRPC6 (Poteser et al., 2006; Ma et al., 2016). Additional evidence pointing to the Orai1 channel as the target of PPC is provided below.

### ROS and Inactivation of Orai1 Channels

In the present study, we confirmed that ROS production increases in response to PPC and found that inhibition of SOC currents can be overcome by administration of the general antioxidant NAC, which reacts with hydroxyl radicals and H_2_O_2_ (Aruoma et al., 1989), suggesting that PPC inhibits SOCs via ROS. MitoKATP channel opening has been shown previously to modulate the mitochondrial redox state and mitochondrial ROS production (Liu et al., 1998; Pasdois et al., 2008; González et al., 2010).

In addition to their well-studied involvement in pathological processes, ROS are also important for normal physiological phenomena (Dröge, 2002), including preconditioning (González et al., 2010). ROS have been shown to reduce Orai1 channel activity in T lymphocytes and Orai1 redox sensitivity has been shown to depend mainly on the extracellular reactive Cys^195^ locus, which is present in Orai1 but absent in Orai3 (Bogeski et al., 2010; Saul et al., 2016), though both isoforms are expressed in cardiomyocytes (Saliba et al., 2015). Meanwhile, Orai2 has an extremely low level of expression in heart tissues (Saliba et al., 2015). Together, such findings support a tentative conclusion that Orai1 channels are the primary, if not the sole, targets of ROS during PPC.

### NCX and Ca^2+^-Dependent Inactivation of SOCs

Because SOCs are strongly inactivated by Ca^2+^ (Prakriya and Lewis, 2015), the extent and duration of Ca^2+^ entry through SOCs depend on [Ca^2+^]_i_ in their microdomains. Ca^2+^-dependent inactivation of SOCs develops over a period of seconds (Zweifach and Lewis, 1995). Among the three Orai isoforms that have been characterized, only Orai1 shows significant Ca^2+^-dependent inactivation (Lis et al., 2007). In our experiments, SOC currents remained active when cardiomyocytes were continuously held at −80 mV. However, when voltage ramps were applied, currents were inactivated within seconds, an effect that could be prevented by Ca^2+^ buffering, consistent with the involvement of Orai1 channels in the generation of SOC currents.

The present finding showing that NCX blockade with Ni^2+^ produced partial restoration of SOC currents in Tg-treated non-PPC cardiomyocytes suggests that the Ca^2+^ that inactivates SOCs upon ramp application is likely provided via NCX operating in the reverse mode (Khananshvili, 2014). Ca^2+^ influx via NCX is expected to inactivate Orai1 channels in their proximity, providing a functional link between NCX and SOCs. In PPC cardiomyocytes, we found that Tg-generated currents were restored by Ni^2+^, suggesting that NCX also plays a role in the inactivation of SOCs by PPC. The formation of a complex between Orai1 and NCX in the plasma membrane observed in our co-immunoprecipitation experiments further supports the notion that these proteins may be associated in a cardiac signaling complex promoting inactivation of SOCE during PPC. Because SOC inactivation reduces Ca^2+^ influx into cardiomyocytes, it could help to attenuate Ca^2+^ overload and thereby provide protection from I/R damage. In agreement with this possibility, inhibition of NCX has been shown to abolish ischemic tolerance in the heart induced by either the volatile anesthetic sevoflurane or ischemic preconditioning (Bouwman et al., 2006; Castaldo et al., 2017).

### Mitochondria Play a Role in Ca^2+^-Dependent Inactivation of Orai1 Channels

Our experiments suggest that a decreased Ca^2+^ buffering capacity by mitochondria during PPC contributes to the promotion of SOC inactivation. Mitochondria are active participants in intracellular Ca^2+^ signaling and oppose slow SOC inactivation by buffering Ca^2+^ and, thus, competing with SOC inactivation sites for Ca^2+^ (Gilabert and Parekh, 2000; Prakriya and Lewis, 2015). In non-excitable cells, mitochondria located near SOCs at the plasma membrane take up considerable amounts of Ca^2+^ through the mitochondrial Ca^2+^ uniporter and regulate Ca^2+^ microdomains (Hoth et al., 1997; Gilabert and Parekh, 2000; Gilabert et al., 2001). Consistent with this role of mitochondria, it has been reported that Ca^2+^ influx through SOCs in Jurkat cells is reduced when mitochondrial buffering capacity is compromised by mitochondrial membrane depolarization following Dzx-induced mitoKATP channel opening (Holmuhamedov et al., 1999, 2002; Valero et al., 2008) or when mitochondria are in a de-energized state (Hoth et al., 1997, 2000).

The use of an energizing cocktail solution in the patch pipette delays and attenuates slow SOC inactivation (Gilabert and Parekh, 2000). In PPC cardiomyocytes, we noted that with use of a similar pipette solution, SOC currents recovered from inactivation when held at −80 mV, even with the application of voltage ramps. Therefore, it is likely that mitochondria are also essential for SOCE activation in this preparation. In agreement with this possibility, mitochondria have been reported to occupy at least 30% of the cell volume in adult cardiomyocytes, and a significant fraction of mitochondria is located in the subsarcolemmal region (Piquereau et al., 2013), where they are able to interact with membrane channels. In fact, previous work has shown that mitochondria help to shape Ca^2+^-dependent inactivation of Cav1.2 channels in adult cardiomyocytes (Sánchez et al., 2001).

### Physiological Significance of PPC-Induced Inhibition of SOCs

The SOC inhibitor glucosamine (Hunton et al., 2002; Nagy et al., 2006; Collins et al., 2013) has been shown to provide protection against I/R injury (Liu et al., 2006), suggesting that SOCs play a role in Ca^2+^ overload (Collins et al., 2013). A potential role for SOCs in I/R is further supported by the observation that SR Ca^2+^ contents are markedly depleted in intact hearts subjected to I/R (Valverde et al., 2010). The ensuing SR Ca^2+^ depletion would be expected to activate SOCE.

## Conclusion

The main findings of this work are that PPC results in a large inhibition of SOC currents upon depletion of SR, and that this effect is due to ROS and Ca^2+^-dependent channel inactivation. Additionally, we showed that SOC currents can be restored by maintaining mitochondria in an energized state, which indicates that mitochondria play a key role in these observations, and that the NCX contributes as a source of channel-inactivating Ca^2+^. Hence, this study suggests that PPC mediated protection against I/R damage may be explained, in part, by decreased SOCE as a result of the largely abrogated influx of Ca^2+^ through SOCs. Although the present experiments were performed in single cells, it is plausible that PPC would produce similar changes in intact hearts. Future research is needed to test this prediction directly. Given the clinical importance of protecting the heart from I/R damage, a detailed understanding of the cellular mechanisms underlying PPC is relevant to the development of cardioprotective therapies.

## Data Availability Statement

The datasets generated for this study are available on request to the corresponding author.

## Ethics Statement

The animal study was reviewed and approved by the Division of Laboratory Animal Units, CINVESTAV.

## Author Contributions

JS designed the study. JS, RS, EC, and MG planned the experiments. RS performed the patch-clamp experiments and ROS measurements. EF and MG performed the Ca^2+^ experiments. EC performed the co-immunoprecipitation experiments. AH assisted with the isolation of cardiomyocytes. JS and MG contributed reagents, material, and acquired financial support for the project. JS analyzed the data and wrote the manuscript. All authors read and approved the final manuscript.

## Conflict of Interest

The authors declare that the research was conducted in the absence of any commercial or financial relationships that could be construed as a potential conflict of interest.

## Supplementary Material

FIGURE S1 Pharmacological preconditioning and cardioprotection. **(A,B)** Cross-sections of isolated ventricles under conditions indicated below panels. Light areas represent areas of infarction after severe ischemia.

FIGURE S2 Pharmacological preconditioning and ROS production. The graph shows mean values (±SEM) of ROS production in control (open circles, *n* = 17) and PPC cardiomyocytes (filled circles, *n* = 10) as a function of time. ^∗^*p* < 0.05, ^∗∗^*p* < 0.01, ^∗∗∗^*p* < 0.001.
