# Supplementary figures and images for: Pharmacological Preconditioning Using Diazoxide Regulates Store-Operated Ca2 + Channels in Adult Rat Cardiomyocytes
Source: Front Physiol. 2020 Jan 14;10:1589. doi: 10.3389/fphys.2019.01589 (PMC6972595; doi:10.3389/fphys.2019.01589)

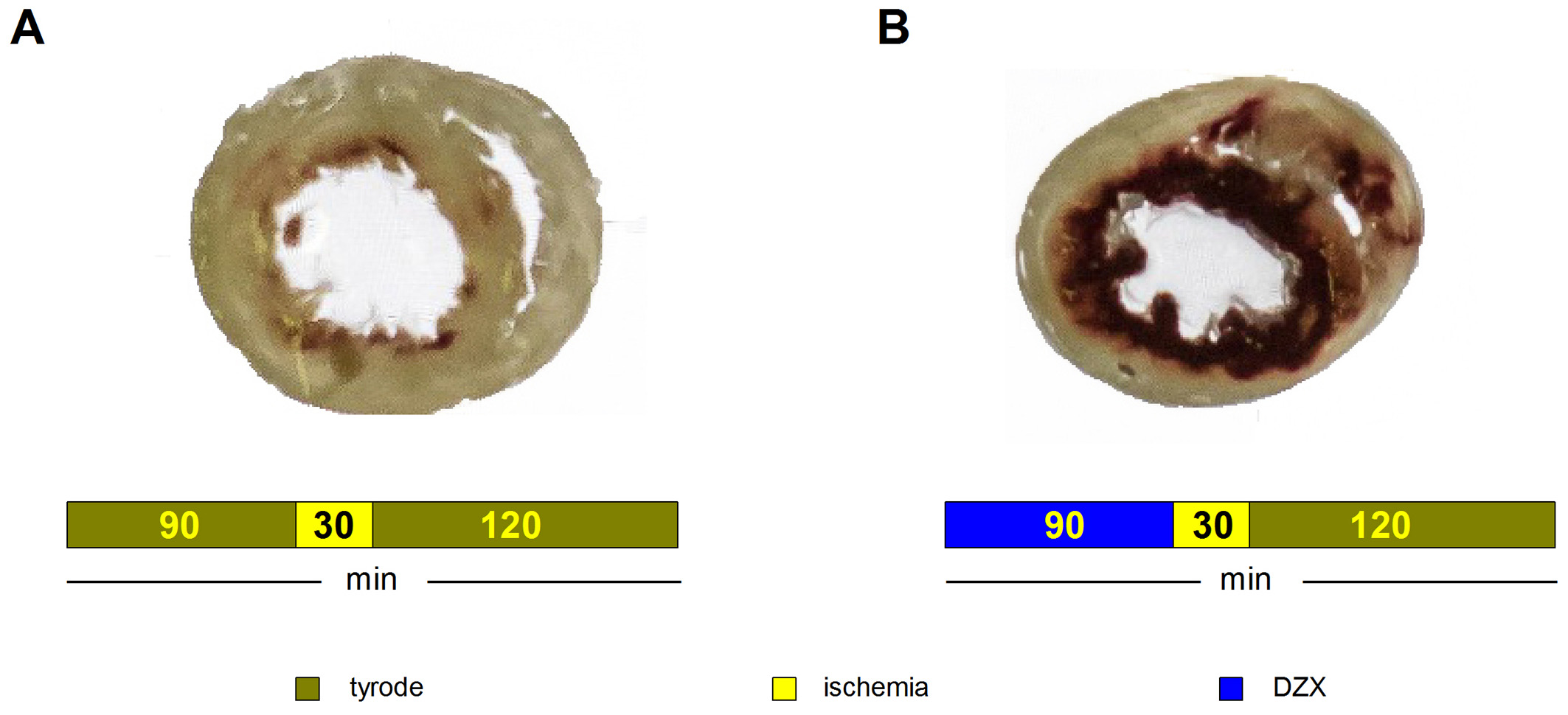

Supplement: FIGURE S1 — Pharmacological preconditioning and cardioprotection. (A,B) Cross-sections of isolated ventricles under conditions indicated below panels. Light areas represent areas of infarction after severe ischemia. [file Image_1.JPEG]

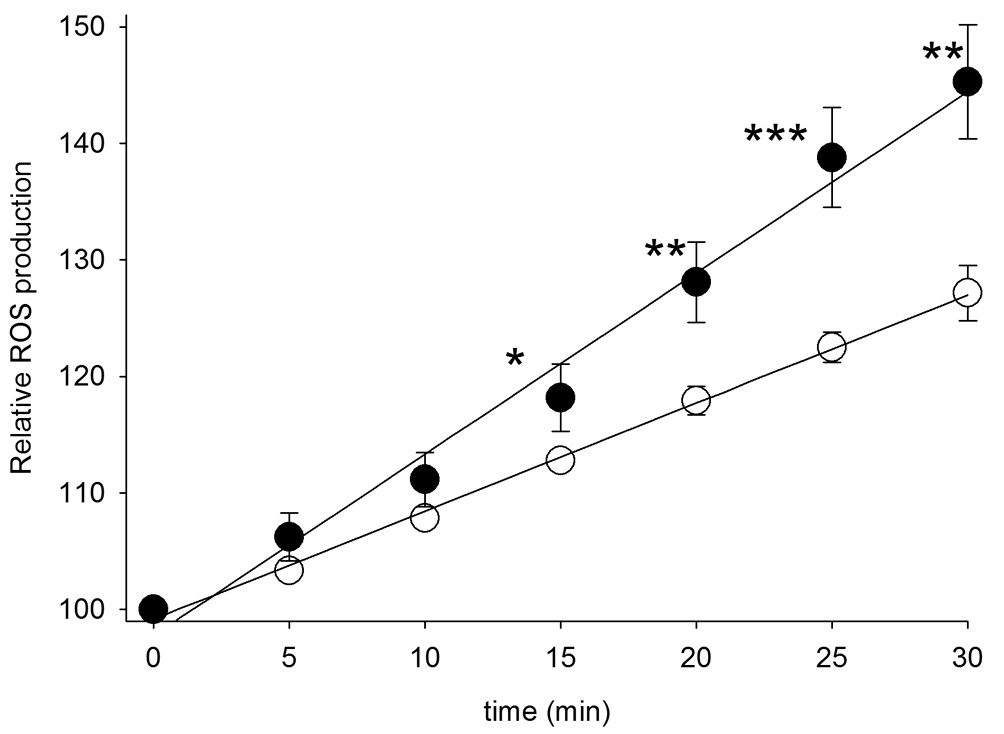

Supplement: FIGURE S2 — Pharmacological preconditioning and ROS production. The graph shows mean values (±SEM) of ROS production in control (open circles, n = 17) and PPC cardiomyocytes (filled circles, n = 10) as a function of time. ∗p < 0.05, ∗∗p < 0.01, ∗∗∗p < 0.001. [file Image_2.JPEG]
